# Production of SARS-CoV-2 Virus-Like Particles in Insect Cells

**DOI:** 10.3390/vaccines9060554

**Published:** 2021-05-26

**Authors:** Youjun Mi, Tao Xie, Bingdong Zhu, Jiying Tan, Xuefeng Li, Yanping Luo, Fei Li, Hongxia Niu, Jiangyuan Han, Wei Lv, Juan Wang

**Affiliations:** 1 Lanzhou Center for Tuberculosis Research and Gansu Provincial Key Laboratory of Evidence Based Medicine and Clinical Translation, School of Basic Medical Sciences, Lanzhou University, Lanzhou 730070, China; miyoujun@126.com (Y.M.); 13643819274@163.com (T.X.); lf@lzu.edu.cn (F.L.); niuhx@lzu.edu.cn (H.N.); hanjy17@lzu.edu.cn (J.H.); lvw18@lzu.edu.cn (W.L.); wangj19@lzu.edu.cn (J.W.); 2 Institute of Immunology, School of Basic Medicine, Lanzhou University, Lanzhou 730070, China; tanjy@lzu.edu.cn (J.T.); luoyp@lzu.edu.cn (Y.L.); 3 Institute of Combined Western and Chinese Traditional Medicine, Lanzhou University, Lanzhou 730070, China; lixuefeng@lzu.edu.cn

**Keywords:** SARS-CoV-2, virus-like particles, insect cells

## Abstract

Coronavirus disease (COVID-19) causes a serious threat to human health. Virus-like particles (VLPs) constitute a promising platform in SARS-CoV-2 vaccine development. In this study, the E, M, and S genes were cloned into multiple cloning sites of a new triple expression plasmid with one p10 promoter, two pPH promoters, and three multiple cloning sites. The plasmid was transformed into DH10 Bac^TM^ *Escherichia coli* competent cells to obtain recombinant bacmid. Then the recombinant bacmid was transfected in ExpiSf9^TM^ insect cells to generate recombinant baculovirus. After ExpiSf9^TM^ cells infection with the recombinant baculovirus, the E, M, and S proteins were expressed in insect cells. Finally, SARS-CoV-2 VLPs were self-assembled in insect cells after infection. The morphology and the size of SARS-CoV-2 VLPs are similar to the native virions.

## 1. Introduction

Coronavirus disease 2019 (COVID-19), caused by the severe acute respiratory syndrome coronavirus-2 (SARS-CoV-2), went on to ravage the world and caused the biggest pandemic in the 21st century. Compared with severe acute respiratory syndrome coronavirus (SARS-CoV) and Middle East respiratory coronavirus (MERS-CoV), severe acute respiratory syndrome coronavirus-2 (SARS-CoV-2) spreads faster with an infection index of about 2.6 [1,2,3]. As of 6 April 2021, more than 130.45 million people worldwide have been infected with the SARS-CoV-2, of whom more than 2.84 million have died [4]. At present, there are no specific drugs for COVID-19, many efforts have focused on neutralizing antibody and vaccine development [5,6,7,8,9]. Vaccines are the most economical and effective means to prevent and control infectious diseases [10]. There is an urgent need to develop efficacious SARS-CoV-2 vaccines against SARS-CoV-2.

Substantial efforts are being made to develop vaccines against SARS-CoV-2. The World Health Organization estimates that there are about 250 vaccines under development [11]. Coronavirus vaccines generally fall into one of the following types: inactive or live-attenuated viruses, protein-based, virus-like particles (VLPs), viral vectors, and nucleic acid vaccines [12]. Inactivated viruses or live attenuated viruses require a large number of viruses to be cultured under biosafety level 3 (BSL3) conditions, and extensive safety testing is required, but the process is expensive, laborious, and has a high safety risk. Protein-based subunit vaccines have poor immunogenicity due to incorrect folding of the target protein or poor display to the immune system, and they require the addition of an adjuvant to induce a strong immune response. Nucleic acid vaccines cannot enter cells efficiently, need to be electroporated after injection, their mRNA is not very stable, and multiple inoculations are required [13]. As a specific type of subunit vaccine, VLPs can mimic the natural morphology and structure of viruses [14]. Because of VLPs strong immunogenicity, ability to elicit protective neutralizing antibodies, and reliable safety, VLPs are strong candidates for vaccines design. At present, several vaccines based on VLPs are commercially available, these include human papillomavirus (HPV) vaccine and hepatitis B vaccine (HBV) [15,16,17,18].

SARS-CoV, SARS-CoV-2, and MERS-CoV all belong to Betacoronaviruses (βCoVs) and have a similar structure [1]. According to previous studies, the composition of SARS-CoV VLPs and MERS-CoV VLPs required the E, M, and S proteins expression in the cells [19,20]. Similar to other βCoVs, the 3′ end of the SARS-CoV-2 genome encodes 4 main structural proteins, including the spike (S) protein, the envelope (E) protein, the membrane (M) protein, and the nucleocapsid (N) protein [21]. We speculate that SARS-CoV-2 VLP also consists of the E, M, and S proteins. In this study, a triple expression plasmid that expresses the E, M, and S proteins was constructed. The Bac-to-Bac baculovirus insect expression system was used to achieve the expression of the E, M, and S proteins in ExpiSf9^TM^ insect cells. Eventually, the E, M, and S proteins self-assemble to form VLPs in the cells.

## 2. Materials and Methods

### 2.1. Cell Lines

ExpiSf9^TM^ insect cells were presented by Mr. Ru Yi from the State Key Laboratory of Lanzhou Institute of Veterinary Medicine, Chinese Academy of Agricultural Sciences. ExpiSf9™ is a cell line that is adapted to high-density suspension growth and has a doubling time of approximately 24 h during log phase growth. ExpiSf9™ cells were maintained as suspension cultures in flasks with serum-free ExpiSf™ CD Medium (Gibco, USA) at 27 °C with stirring at a speed of 125 rpm. Cell density was determined by microscopically counting the number of cells, and cell viability was judged by trypan blue dye exclusion.

### 2.2. Construction of the EMS Triple Expression Plasmid

The dual expression plasmid pFastBacDual (Invitrogen, USA) was purchased from Thermo Fisher Scientific China Co., Ltd. In order to obtain a single recombinant baculovirus that expressed the M, E, and S proteins, a new triple expression vector was generated. In brief, a new SV40 poly A tail, a new pPH promoter, and a *NdeI* restriction site were inserted into the *EcoRI* and *HindIII* restriction sites after pPH promoter of pFastBacDual. The new triple expression vector has one p10 promoter and two pPH promoters. For expression of the E, M, and S proteins, the codon optimized E, M, and S genes of SARS-CoV-2 (GenBank accession No. MN908947.3) were cloned into the triple expression vector. The E gene cloned into the double *KpnI* and *XhoI* restriction sites under the control of the p10 promoter, the M gene inserted into the *BamHI* and *EcoRI* restriction sites under the control of the pPH promoter, and the S gene cloned into the double *NdeI* and *HindIII* restriction sites under the control of other pPH promoter. Finally, the triple expression plasmid named EMS was generated and verified by DNA sequencing (BGI, China).

### 2.3. Generation of Recombinant Baculovirus

Recombinant baculovirus was generated by using a Bac-to-Bac expression system (Invitrogen, USA) according to the manufacturer’s instructions. Briefly, the EMS plasmid was transformed into Top10 competent (Transgen, China). After re-extracting the EMS plasmid, it was transformed into DH10 Bac^TM^ *E. coli* competent (Invitrogen, USA). White colonies were screened in LB media containing the antibiotics gentamicin (7μg/mL), tetracycline (10 μg/mL), kanamycin (50 μg/mL), X-Gal (5-bromo-4-chloro-3-indolyl-β-D-galactopyranoside), and IPTG (isopropyl-β-D-thiogalactopyranoside). After two cycles of white colony screening, recombinant bacmid DNA were isolated. ExpiSf9™ cells were subcultured until the cells reached a density of approximately 5 × 10^6^–10 × 10^6^ viable cells/mL and ≥90% viability, transfection of 62.5 × 10^6^ cells with 2.5 μg bacmid DNA. Transfected ExpiSf9^TM^ cells were cultured at 27 °C and 125 rpm, until the cytopathic rate reached 30%, the cell supernatant was collected to obtain the recombinant baculovirus. PCR was used to verify the genes of interest in the recombinant bacmid. The primers for PCR are shown in Table 1.

### 2.4. Preparation of EMS VLPs 

ExpiSf9^TM^ cells were incubated until the cells reached a density of approximately 5 × 10^6^–7 × 10^6^ viable cells/mL and ≥80% viability, ExpiSf™ Enhancer (Invitrogen, USA) was added to the cell culture. Twenty-four hours after the addition of ExpiSf™ Enhancer, cells were infected with baculovirus in a volume of 50:1. Cells were collected on day 4 by centrifugation at 3000× *g* for 5 min, freeze-thawed repeatedly for 3 times, and the pellet was discarded after centrifugation at 8000× *g* for 30 min. The supernatant was ultra-centrifuged at 100,000× *g* for 1 h at 4 °C, and the pellets were resuspended in phosphate-buffered saline (PBS) at 4 °C overnight. EMS VLPs were put through a 30%–40%–50% discontinuous sucrose gradient at 100,000× *g* for 2 h at 4 °C. The white bands between 30% and 40% were collected and diluted with PBS and pelleted at 100,000 *g* for 1 h at 4 °C. The VLPs were collected and resuspended in PBS overnight at 4 °C. EMS VLPs were stored at −80 °C for the following analysis.

### 2.5. Western Blot Analysis

The VLPs were characterized by Western blot analysis and electric microscopic observation. For Western blot analysis, VLPs samples were subjected to SDS-PAGE using a 10% gel, followed by transfer to polyvinylidene difluoride (PVDF) membranes. The PVDF membranes were then blocked with TBST (10 mM Tris-HCl, 150 mM NaCl, 0.5% Tween 20) containing 5% skim-milk powder. The S protein of VLPs was detected by Western blotting, using an anti-SARS-CoV-2 S polyclonal rabbit antibody (Sino Biological, China). The expression of the E and the M protein were also verified by polyclonal antibodies (data not shown). Alkaline phosphatase-conjugated goat-anti-rabbit IgG (ImmunoWay, Plano, TX, USA) were used as the secondary antibodies to label the protein bands.

### 2.6. Electron Microscopy

For transmission electron microscopy, 5 μL VLPs samples were applied onto a carbon-coated film. Two minutes later, the samples were removed with filter paper. Then, 8 μL of 1% phosphotungstic acid was applied onto the grid, and the samples were stained for 60 s. The staining solution was removed with filter paper, and the grid was dried for 30 min at room temperature. After being stained, the sample was observed using a FEI Talos F200C transmission electron microscope (FEI, Czech Republic) at 200 kV and 100–200 k-fold magnification.

## 3. Results

### 3.1. Construction of EMS Triple Expression Plasmid

**Figure 1:**
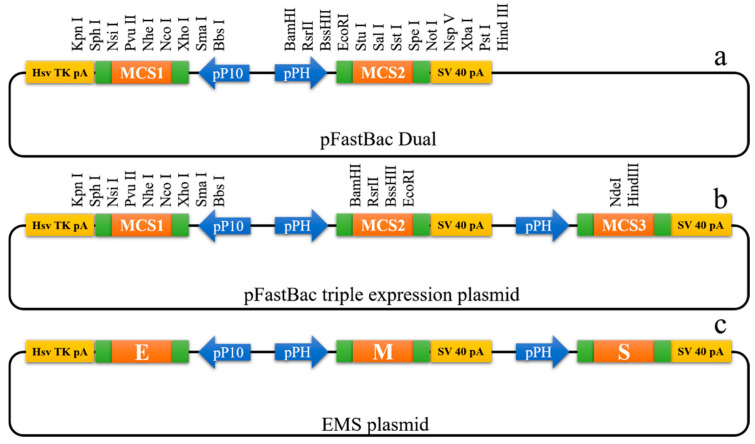
Construction of EMS plasmid. (**a**) pFastBac Dual plasmid. MCS, multiple cloning sites; SV 40 pA, SV 40 poly A tail; Hsv TK pA, herpes simplex virus thymidine kinase polyadenylation signals; pPH, pPH promoter; pP10, pP10 promoter. (**b**) Construction of pFastBac triple expression plasmid with one p10 promoter and two pPH promoters. (**c**) Construction of EMS plasmid for expression of the E, M, and S proteins.

To express the SARS-CoV-2 E, M, and S proteins in insect cells, a new triple expression vector was constructed. The triple expression vector contained one pP10 promoter, two pPH promoters, and three multiple cloning sites (Figure 1b). Then, the SARS-CoV-2 E, M, and S genes were cloned into multiple cloning sites, respectively, to generate EMS plasmid (Figure 1c).

### 3.2. Generation of Recombinant Bacmid DNA

**Figure 2:**
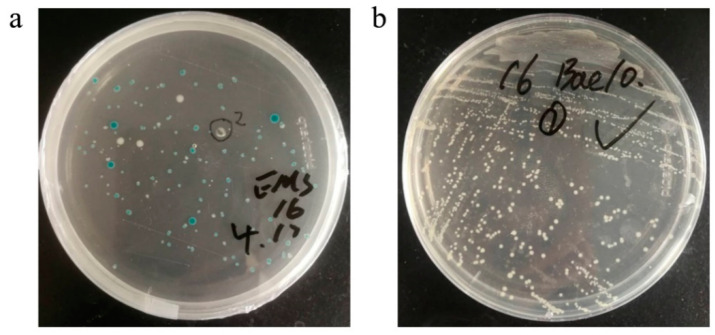
Transformation and secondary screening. (**a**) The EMS vector was transformed into DH10 Bac^TM^ competent cells. (**b**) Secondary screening after transformation.

**Figure 3:**
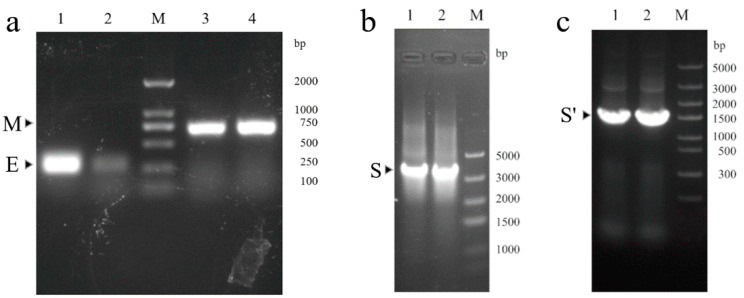
Analysis of recombinant bacmid DNA by PCR. Two colonies were picked for PCR. (**a**) 1,2, identification of the E gene by PCR; 3,4, identification of the M gene by PCR. (**b**) 1,2, identification of the S gene by PCR. (**c**) 1,2, identification of S’ DNA fragment by PCR.

The EMS recombinant plasmid was transformed into DH10 Bac^TM^ *E. coli* competent cells. Blue and white colonies were visible on the LB screening plate after 48 h (Figure 2a). White colonies were picked for the second screening to obtain positive colonies that contained the recombinant bacmid (Figure 2b). PCR analysis was used to verify the E, M, and S genes in the recombinant bacmid. The target bands could be obtained at the corresponding positions (Figure 3). 

### 3.3. Recombinant Baculovirus Production

**Figure 4:**
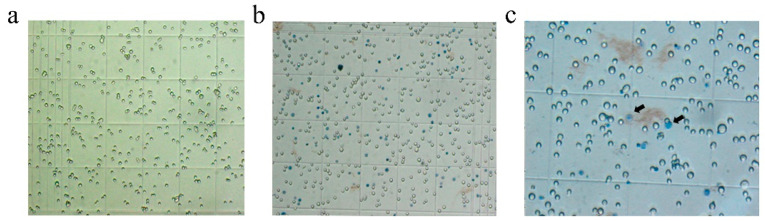
Cytopathic signs observed in ExpiSf9^TM^ cells. (**a**) ExpiSf9^TM^ cells (×10). (**b**) Transfected ExpiSf9^TM^ cells (×10). (**c**) ExpiSf9^TM^ cells with visible signs of virus infection (× 40).

After confirmation that the recombinant bacmid contains the E, M, and S genes, transfect ExpiSf9™ insect cells to produce recombinant baculovirus. Following the recombinant bacmid was added to the cells, incubate the cells until visible signs of virus infection. Swollen cells with enlarged nuclei indicated that a cell was infected by the baculovirus (Figure 4). After trypan blue staining, the cell death rate was increased. The recombinant baculovirus was collected from the cell culture medium when the cells’ characteristics appeared typical of late to very late infection.

### 3.4. Production and Characterization of SARS-CoV-2 VLPs

**Figure 5:**
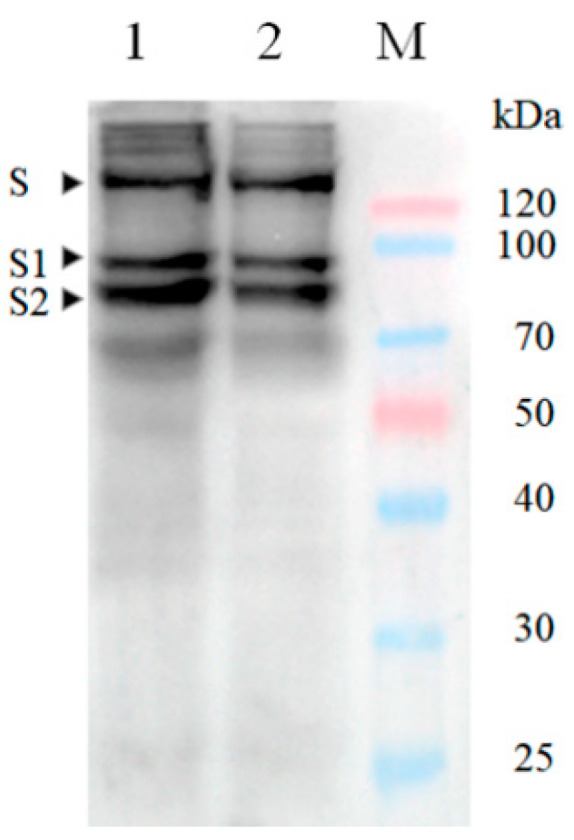
Western blotting identification of SARS-CoV-2 S protein expressed on VLPs. 1,2, identification of SARS-CoV-2 S protein with S polyclonal antibody; S, spike protein; S1, S1 fragment of spike protein; S2, S2 fragment of spike protein; M, Protein maker.

**Figure 6:**
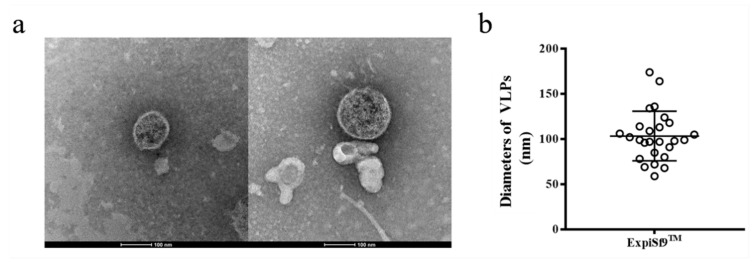
Morphological evaluation of SARS-CoV-2 VLPs. (**a**) TEM pictures of prepared VLPs. Scale bar = 100 nm. (**b**) Quantification of diameters of SARS-CoV-2 VLPs formed from ExpiSf9^TM^ cells.

To produce SARS-CoV-2 VLPs in insect cells, ExpiSf9 ^TM^ cells were infected with recombinant baculovirus. Ninety-six hours after infection, the cells were harvested, and SARS-CoV-2 VLPs were purified by sucrose gradient centrifugation. Approximately 500 μg VLPs could be harvested from 100 mL of culture. To confirm that the S protein was incorporated within the VLPs, purified VLPs were analyzed by Western blot using SARS-CoV-2 S protein polyclonal antibody. The result showed that VLPs contained the SARS-CoV-2 S proteins (Figure 5). The morphology of SARS-CoV-2 VLPs was investigated by electron microscopy. The SARS-CoV-2 VLPs exhibited spheriform structures, and the average diameter of the VLPs fell around 103.30 ± 27.44 nm (Figure 6). These results indicate that SARS-CoV-2 VLPs autonomously assemble in insect cells infected with recombinant baculovirus, and that they are structurally similar to the native virions [22].

## 4. Discussion

Although some countries have successfully controlled the domestic spread of COVID-19, in the face of ongoing death and destruction caused by the SARS-CoV-2 virus, there is still an urgent need for developing vaccines against the spread of the virus. At present, SARS-CoV-2-related traditional vaccines and new generation vaccines are under development, but there is no effective vaccine approved for use. Because of their unique advantages, VLPs have been use as vaccine platform. 

SARS-CoV-2, SARS-CoV, and MERS-CoV have similar virus structures, which consist of four structural proteins: N, E, M, S [21]. The M, E, and S proteins self-assembled to form SARS-CoV VLPs when co-transfecting the E, M, and S baculoviruses in Sf9 insect cells [19]. SARS-CoV VLPs are immunogenic and can cause strong SARS-CoV VLPs-specific humoral and cellular immune responses in mice [23]. Similarly, the co-infection of MERS CoV E, M, and S recombinant baculoviruses in insect cells produces VLPs with a similar morphological signature to the native virions [20]. MERS-CoV VLPs are immunogenic and are able to elicit robust levels of specific humoral and cell-mediated immunity in rhesus monkeys. MERS-CoV VLPs with alum adjuvant induced high titer virus neutralizing antibodies and triggered T helper 1 cells (Th1)-mediated immunity in vaccinated rhesus monkeys [20]. Recently, mammalian expression systems have been utilized to construct SARS-CoV-2 VLPs. For example, SARS-CoV-2 VLPs were produced by plasmid-driven transfection of viral structural proteins in HEK-293T cells or VeroE6 cells [24]. And HEK-293T cells are commonly used to produce SARS-CoV-2 VLPs [24,25,26]. Mammalian cells produce fewer of the VLPs with higher production cost compared with other systems [27]. In addition, HEK-293T cells and VeroE6 cells are adherent cells, and the upstream scale-up of culturing adherent cells is generally complex [28].

The baculovirus–insect cell system is especially suitable for virus-like particle production because this system allows more than one gene to be expressed in a cell. This system has many advantages including safety, high levels of protein expression, eukaryotic posttranslational modifications, and scalability. The baculovirus–insect cell system has been widely used for the production of a wide variety of VLPs [29,30,31,32,33,34]. In this study, a new triple expression vector was constructed, after which the E, M, and S genes were cloned into the triple expression vector to generate EMS plasmid. The EMS vector was transformed into DH10Bac^TM^ competent cells, and the recombinant bacmid was obtained after screening twice. Subsequently, the recombinant bacmid was transfected in ExpiSf9^TM^ insect cells to obtain recombinant baculovirus. After ExpiSf9^TM^ cells infection with the recombinant baculovirus, the E, M, and S proteins expressed in cells formed VLPs by self-assembly. To our knowledge, the successful construction of SARS-CoV-2 VLPs via an insect expression system has not yet been reported. In the future, humoral and cellular immunogenicity of SARS-CoV-2 VLPs in animal models will be further evaluated. In addition, VLPs can also be used to study the pathogenesis of COVID-19.

## Figures and Tables

**Table 1:** Analysis of recombinant bacmid DNA by PCR.

Genes	Size of PCR Product	Primers	Sequence (5′-3′)
E	228 bp	EF	ATGTACTCATTCGTTTCGGA
		ER	TTAGACCAGAAGATCAGGAACTC
M	669 bp	MF	ATGGCAGATTCCAACGGTA
		MR	TTACTGTACAAGCAAAGCAATATT
S	3819 bp	SF	ATGTTTGTTTTTCTTGTTTTATTG
		SR	TTATGTGTAATGTAATTTGACTCCTTT
S’	1486 bp	S’F	AAACACGCTTGTTAAACAAC
		M13R	CAGGAAACAGCTATGAC

## Data Availability

The data presented in this study are available on request from the corresponding author.
